# Action in Perception: Prominent Visuo-Motor Functional Symmetry in Musicians during Music Listening

**DOI:** 10.1371/journal.pone.0138238

**Published:** 2015-09-30

**Authors:** Iballa Burunat, Elvira Brattico, Tuomas Puoliväli, Tapani Ristaniemi, Mikko Sams, Petri Toiviainen

**Affiliations:** 1 Finnish Centre for Interdisciplinary Music Research, Department of Music, University of Jyväskylä, Finland; 2 Department of Mathematical Information Technology, University of Jyväskylä, Jyväskylä, Finland; 3 Center for Music in the Brain (MIB), Department of Clinical Medicine, Aarhus University, Aarhus, Denmark; 4 Department of Neuroscience and Biomedical Engineering, Aalto University School of Science, Espoo, Finland; 5 Cognitive Brain Research Unit (CBRU), Institute of Behavioral Sciences, University of Helsinki, Helsinki, Finland; 6 Advanced Magnetic Imaging (AMI) Centre, Aalto University School of Science, Espoo, Finland; UNLV, UNITED STATES

## Abstract

Musical training leads to sensory and motor neuroplastic changes in the human brain. Motivated by findings on enlarged corpus callosum in musicians and asymmetric somatomotor representation in string players, we investigated the relationship between musical training, callosal anatomy, and interhemispheric functional symmetry during music listening. Functional symmetry was increased in musicians compared to nonmusicians, and in keyboardists compared to string players. This increased functional symmetry was prominent in visual and motor brain networks. Callosal size did not significantly differ between groups except for the posterior callosum in musicians compared to nonmusicians. We conclude that the distinctive postural and kinematic symmetry in instrument playing cross-modally shapes information processing in sensory-motor cortical areas during music listening. This cross-modal plasticity suggests that motor training affects music perception.

## Introduction

Within-modality neuroplasticity has been investigated extensively in the sensory and motor modalities, demonstrating the adaptive (or maladaptive [1] capabilities of the human brain to shape its processing of a sensory stimulus or to perform motor acts after repeated sensory exposure or action [2,3]). Comparing brain function and anatomy of musically trained and untrained individuals is ideal for studying neuroplasticity because of a large difference between groups in time spent with music-related activities.

Musical activities, such as playing an instrument from a musical score, involve cross-modal orchestration of auditory, visual, somatomotor, and cognitive processes [4,5]. Nevertheless, studies showing plasticity of brain function and structure associated with intensive musical training have thus far focused on within-modality brain measures. Early musical training has been shown to correlate with stronger auditory-cortical representations of piano vs. pure tones in pianists, supported by anatomical enlargements of the Heschl’s gyrus [6,7]. The left-hand fingers of string players exhibit more extensive contralateral somatosensory cortical representations than those of nonmusicians. This effect is stronger for those string players who began musical practice at an early age [8,9]. Musicians also show more anatomical symmetry in cortical motor regions compared to controls [10]. The linking of brain anatomy and acquired sensorimotor skills is further evident in consistent within-musician differences observed in the right-left precentral gyrus depending on the instrument played [11].

These findings have led to the hypothesis that functional reorganization may cause structural adaptation [12–14]. Thus, the asymmetrical hand-motor requirements may drive the enlarged left-hand somatosensory representation in violin players. Even when limited to fifteen months in childhood [14], musical training drives an increase in grey matter for areas involved in motor, auditory, and visuo–spatial processing [15]. Similarly, an increase in cerebellar volume, presumably in response to the intensity of instrumental practice in musicians [16], suggests structural reorganization induced by long-term motor and cognitive demands derived from intense music-related auditory and motor practice. Furthermore, the size of the anterior corpus callosum (CC), which mainly connects motor areas, is enlarged in individuals with an early commencement of musical training [17,18].

The aforementioned findings motivate this study, in particular those on enlarged anterior callosum in musicians, and on enhanced somatosensory left-hand finger representation in string players. It could be assumed that morphological differences in CC are reflected in the interhemispheric functional connectivity in musicians. Several morphometric studies suggest that callosal volume predicts interhemispheric transfer capacity [19,20] and there exists evidence of a positive correlation between callosal area and the amount of fibres crossing through supporting this view [21]. However, the literature is not in agreement regarding a positive correlation between callosal size and interhemispheric transfer capacity [22–27].

Furthermore, differences in interhemispheric information transfer deriving from musical training have been only marginally investigated. While Patston and others [28] found an unusual symmetry in musicians’ (mostly pianists) interhemispheric transfer speed of visual information compared to nonmusicians, previous studies have not investigated this phenomenon using musical stimulation, and have employed paradigms with controlled simple stimuli thus not allowing generalization to real life situations. Increased communication between hemispheres may extend beyond the somatosensory and motor system to other modalities that are relevant to music processing.

Here we studied the relationship between musical training, callosal volume, and interhemispheric functional symmetry in brain activity measured using functional magnetic resonance imaging (fMRI) during continuous listening to natural music. By interhemispheric functional symmetry we refer to the voxel-mirrored homotopic connectivity [29] as measured by the coactivation of homotopic (i.e., topographically matched) brain areas. Our approach consisted of three stages: (a) morphometry of participants’ callosa was computed to examine a possible relationship between callosal volume and group membership; (b) symmetry indices were estimated for all voxels in the brain; and (c) significant differences between groups (musicians vs. nonmusicians and keyboard vs. string players) were assessed. We hypothesized that we would find more prominent functional symmetry in musicians, particularly in keyboard players, and particularly within motor-related brain areas. We also expected that this enhanced symmetry be accompanied by an increase in callosal volume.

## Materials and Methods

### Participants

The ethics committee of the Coordinating Board of the Helsinki and Uusimaa Hospital District (Koordinoiva) approved this study with the approval number 315/13/03/00/11. Informed written consent was obtained from all participants. Consent forms are stored in a locked cabinet of NMG Data Repository. Ethics committee approved the form and the procedure. Thirty-six healthy participants with no history of neurological or psychological disorders participated in the fMRI experiment. The participants were screened for inclusion criteria before admission to the experiment (no ferromagnetic material in their body; no tattoo or recent permanent colouring; no pregnancy or breastfeeding; no chronic pharmacological medication; no claustrophobia). The participant pool was selected to be equally divided between musically trained (n = 18) and untrained participants (n = 18, left-handers = 1). The criteria for nonmusicianship was having less than 5 years of music training, not having finished a Music degree in a Music academy, not reporting themselves as musicians, and never earned money for playing. These details were obtained and crosschecked via questionnaires and HIMAB [30] (Helsinki Inventory for Music and Affect Behavior). Both groups were comparable with respect to gender, age distribution, cognitive measures (Processing Speed and Working Memory Index Scores from the WAIS-WMS III [31]), and socioeconomic status (according to Hollingshead’s Four-Factor Index [32]; see Table 1 and Table 2 for demographic data). The musicians’ group was homogeneous in terms of the duration of their musical training, onset age of instrument practice, and amount of years of active instrument playing.

**Table 1:** Demographic information about our sample.

group	N	age	gender	hand	soc-eco status	WAIS-III PSI	active listening (h/week)	passive listening (h/week)	total listening (h/week)
**MUS**	18	28.2±7.8	9F	18R	43.6	116.3	7.5±5.8	10.6±7.5	18.2±11.2
**KEY**	8	26.4±7	4F	8R	37.7	119.8	9.7±6.3	11.5±8.3	21.2±13.4
**STR**	7	28.4±7.9	5F	7R	45.3	110	5.3±1.9	10±7.3	15.3±6.1
**NMUS**	18	29.2±10.7	10F	17R	35.4	115.7	5.3±4.8	7.1±3.9	12.4±6.7

Abbreviations: MUS = musicians, KEY = keyboard players, STR = string players, NMUS = nonmusicians, class = classical, soc-eco = socioeconomic, PSI = Processing Speed Index, WMI = Working Memory Index.

**Table 2:** Specific demographic information about musicians.

group	instrument starting age	instrument playing (years)	instrument practicing (h/week)	musical training (years)	style
**MUS**	8.2±4	21.2±6.2	16.6±11	15±4.7	12 class | 4 jazz | 2 pop
**KEY**	7±2.6	20.1±7.2	15.6±13	14.4±4	5 class | 2 jazz | 1 pop
**STR**	8.3±3.9	21.1±6.2	17.3±12.6	15.9±3.8	6 class | 1 jazz

Abbreviations: MUS = musicians, KEY = keyboard players, STR = string players.

### Stimuli

Three musical pieces were used in the experiment: (a) Stream of Consciousness by Dream Theater; (b) Adios Nonino by Astor Piazzolla; and (c) Rite of Spring (comprising the first three episodes from Part I: Introduction, Augurs of Spring, and Ritual of Abduction) by Igor Stravinsky. These are a progressive rock/metal piece, an Argentinian New Tango, and an iconic 20th century classical work, respectively, thus covering distinct musical genres and styles. All three selected pieces are instrumental and have a duration of about 8 minutes.

### Morphometric analyses of the corpus callosum

Volumetric whole brain segmentation was performed using the FreeSurfer image analysis suite (stable Linux version 5.3.0 released on 15th of May 2013), which is available online at https://surfer.nmr.mgh.harvard.edu/. It provides completely automated parcellation of cortical and subcortical structures previously described [33–35] by assigning a neuroanatomical label to each voxel in an intensity renormalized MRI volume based on probabilistic information estimated from a manually labelled training set [33,35]. The method has been shown to be robust and comparable in accuracy to manual labelling [33,36].

The CC was segmented into five equally spaced regions of interest along the primary eigendirection as per Freesurfer’s default settings (which segments the CC as a 5 mm thick slab, and divides it into 5 segments of equal length). The five regions were posterior, middle posterior, central, middle anterior, and anterior. Following this, the CC was then reorganized in two sections approximating the division used by Lee and others [18]: posterior (comprising posterior, middle posterior, and central) and anterior (comprising middle anterior and anterior). Thus the anterior CC contains interhemispheric fibres of primary somatomotor and other PFC areas, and the posterior part contains those of posterior parietal, temporal, and occipital areas [37]. Mean and standard deviation measures were extracted from posterior and anterior callosal sections.

A significant correlation was found between total callosal volume and total brain volume (*r* = 0.46, *p* < 0.001). Since the cross-sectional area of a 3D object increases as the two/thirds power of the object’s volume [38], relative callosal sizes to the two/thirds power of the total brain volume were used [18].

T-tests were performed to investigate a potential relationship between participants’ callosal volumes and their group membership. We hypothesized musicians’ callosa to be larger than nonmusicians, and keyboardists’ to be larger than string players’. To this end, two directional (right-tailed) t-tests were performed per callosal section to compare (a) musicians vs. nonmusicians, and (b) keyboardists vs. string players.

### fMRI experimental procedure

Participants’ brain responses were acquired while they listened to each of the musical stimuli in a counterbalanced order. For each participant the stimuli loudness was adjusted to a comfortable but audible level inside the scanner room (around 75 dB). In the scanner, participants’ only task was to attentively listen to the music delivered via high-quality MR-compatible insert earphones while keeping their eyes open.

### fMRI scanning and preprocessing

Scanning was performed using a 3T MAGNETOM Skyra whole-body scanner (Siemens Healthcare, Erlangen, Germany) and a standard 20-channel head-neck coil, at the Advanced Magnetic Imaging (AMI) Centre (Aalto University, Espoo, Finland). Using a single-shot gradient echo planar imaging (EPI) sequence thirty-three oblique slices (field of view = 192x192 mm; 64x64 matrix; slice thickness = 4 mm, interslice skip = 0 mm; echo time = 32 ms; flip angle = 75°) were acquired every 2 seconds, providing whole-brain coverage were imaged per participant. T1-weighted structural images (176 slices; field of view = 256x256 mm; matrix = 256×256; slice thickness = 1 mm; interslice skip = 0 mm; pulse sequence = MPRAGE) were also collected for individual coregistration. Functional MRI scans were preprocessed on a Matlab platform using SPM8 (Statistical Parametric Mapping), VBM5 for SPM (Voxel Based Morphometry [39]; Wellcome Department of Imaging Neuroscience, London, UK), and customized scripts developed by the present authors. For each participant, low-resolution images were realigned on six dimensions using rigid body transformations (translation and rotation corrections did not exceed 2 mm and 2° respectively), segmented into grey matter, white matter, and cerebrospinal fluid, and registered to the corresponding segmented high-resolution T1-weighted structural images. These were in turn normalized to the MNI (Montreal Neurological Institute [40]) segmented standard a priori tissue templates using a 12-parameter affine transformation. Functional images were then blurred to best accommodate anatomical and functional variations across participants as well as to enhance the signal-to-noise by means of spatial smoothing using a 8 mm full-width-at-half-maximum Gaussian filter. Movement-related variance components in fMRI time series resulting from residual motion artefacts, assessed by the six parameters of the rigid body transformation in the realignment stage were regressed out from each voxel time series. Following this, spline interpolation was used to detrend the fMRI data, followed by temporal filtering (Gaussian smoothing with kernel width = 4 sec).

### Symmetrization of the brain template

Because the brain is not symmetrical (as manifested by the twisting effect, known as the Yakovlevian torque, and the right frontal and left occipital protrusions, known as petalia), homotopic voxels are not anatomically equivalent in some brain regions. To counterbalance these inherent neuroanatomical asymmetries, and make the claim for homotopic equivalency stronger, participants’ brains were transformed with a spatial mapping. The goal was to create a mirror image of the continuous brain template, where voxel values represent the different intensities of the neural tissue. We thus considered the symmetrization of the brain as an unconstrained nonlinear optimization problem, aimed at minimizing a cost function—the mean squared error (MSE)—between the intensity values of the homotopic voxels of the whole brain template, as shown in Eq 1,

$$\tilde{a}=\underset{a}{argmin}\frac{1}{V(B_{L})}\int_{B_{L}}{\left[b(f_{a}(r(x)))-b(x)\right]}^{2}dx$$ (1)

Here x is any position (x,y,z) in the left hemisphere space $\left(x\in B_{L}\right)$; the function r maps the 3D coordinate point onto its homotopic counterpart (r(x):(x,y,z)→(−x,y,z)); the function b returns the intensity values at the points x; $\tilde{a}$ is the optimal set of parameters for the transformation matrix $f_{a}$which maps the right hemispheric intensity values b(r(x)) onto the left ones b(x), so that the cost function yields the minimum error; $f_{a}(r(x))$is a 5^th^ order polynomial transform. The formula is expressed as the integration of an idealized continuous 3D space. However, in reality we only know the intensity values at the grid points of the brain template, hence the other points are estimated via trilinear interpolation.

The search for the minimum was computationally expensive due to the high number of iterations and the size of the augmented matrix, which required the use of a HP super cluster (taito.csc.fi). The algorithm used for minimizing the objective function was the Nelder-Mead simplex algorithm [41].

Individuals’ brains were symmetrized using the set of parameters that yielded the minimum or stationary point of the cost function. This minimum depended on the point of departure of initial conditions in the parameter space and different initial conditions do not necessarily converge to a minimizer. By randomizing the initial conditions we can reach different stationary points and chose the optimal minimizer.

The differences between the original and symmetrized brain templates were fairly minimal. This can be explained by the smoothing kernel (width = 8 mm) used in the spatial preprocessing of the fMRI data, which would override the potential asymmetries of the brain. The MSE between hemispheres of the original and transformed template were 5.7 mm^2^ and 2.37 mm^2^, respectively.

### fMRI functional symmetry analysis

Brain responses to the three stimuli were concatenated making a total of ~24 minutes worth of data. The rationale behind this was to combine stimuli representing a wide range of musical genres and styles in order to cancel out effects that the specific kinds of music may have on the phenomenon under investigation. The final time series had 702 samples after the 4 first samples of each of the three runs were removed to avoid artefacts due to magnetization effects. Following this, symmetry indices per voxel were computed for all participants’ brains. The symmetry index is the mirrored homotopic connectivity per voxel. It is obtained by correlating each voxel time series with its homotopic counterpart, i.e., correlating the brain with its own flipped image across the midsagittal plane. The results provide for each pair of homotopic voxels a measure of their degree of functional symmetry. Next, symmetry indices were transformed using Fisher’s r-to-Z transformation [42] (see Eq 2) to make their sampling distribution approximately normal.

$$z_{f}=arctan(r)$$ (2)

Significance had to be corrected due to the intrinsic serial correlation of the fMRI time series. To this purpose, we estimated the effective degrees of freedom of the data following a nonparametric permutation-based approach [43] as shown in Eq 3).

$$\frac{1}{df}\approx \frac{1}{N}+\frac{2}{N}\sum \frac{N-j}{N}\rho_{xx}(j)\rho_{yy}(j)$$ (3)

where N is the number of observations, $\rho_{xx}(j)$and $\rho_{yy}(j)$ are the autocorrelations of the pair of homotopic voxel time series at lag j. For each participant the effective degrees of freedom were computed by randomly selecting 1,000 pairs of homotopic voxels as the inputs x and y in Eq 3. Next, estimates from all trials across participants were averaged (mean = 110±9.6), and used to compute the significance of the symmetry index scores by dividing these by the standard error (see Eq 4).

$$z_{corrected}=z_{f}\sqrt{df-3}$$ (4)

Once whole-brain functional symmetry maps were computed and corrected for each participant, directional unpaired two-sample t-tests (alpha = 0.01, one-tailed) were performed on participants’ symmetry indices in order to observe where in the brain each of the groups showed significantly greater symmetry over the other. The resulting spatial maps were corrected for multiple comparisons using the cluster-wise significance approach by Ledberg and others [44]. This is based on a Monte Carlo procedure to assess the null distribution of the cluster sizes (CS) at a particular significance level, from which the critical CS threshold can be selected.

Following Ledberg and others’ method, we first computed the functional symmetry maps for all participants by correlating the brain with its own flipped and phase-scrambled image [45]. The phase-scrambling was done in the volume domain using the 3-D Fourier transform. Once functional symmetry maps were computed, the between-groups t-test statistical map was obtained, from which an estimate of the autocorrelation function (ACF) kernel was computed.

This procedure was repeated to obtain an estimate of the ACF kernel as the average of 10 runs. This averaging decreased the amount of error and led to a more accurate ACF kernel estimate. Next, the ACF kernel was convolved with noise to generate statistical images with the same spatial spectral properties as the resulting t-test maps without containing any signal of interest. We generated 10,000 images from which we derived a probability distribution of cluster sizes above a given threshold, which was subsequently used to estimate the critical cluster size for our data. Finally, the spatial maps resulting from the t-tests were cleaned to retain clusters with a cluster size probability *p* < 0.001 (critical cluster size > 55 voxels).

At this point the resulting spatial maps showed the brain areas that are significantly more symmetrical for one group over the other (i.e., musicians > nonmusicians, nonmusicians > musicians, keyboardists > string players, and string players > keyboardists). Still, the mean symmetry index for a given voxel in group 1 may not be significantly different from zero, even though it may be significantly different from group 2. To ensure that only significantly functional symmetry is retained, t-test spatial maps were further masked with the functional symmetry averaged Fisher Z-map of the group favoured in the right-tailed t-tests at a significant level *p* < 0.0005 (right-tailed). Thus pairs of homotopic voxels whose symmetry indices did not reach significance were discarded.

Anatomical labelling was based on the Automated Anatomical Labelling (AAL [46]) implemented in the MarsBaR toolbox v0.43 (http://marsbar.sourceforge.net) and thus anatomical regions within each cluster were determined. Regions of interest were visually inspected using the MNI structural atlas and the Harvard-Oxford cortical and subcortical atlases implemented in FSL to ensure that the automatic assignment was conforming to the neurological knowledge. The x y z coordinates (in MNI space) of the maximum voxel Z-value within each anatomical region were retrieved and accordingly labelled.

## Results

### Morphometric analysis of the corpus callosum

T-tests results comparing relative CC volume to total brain volume were nonsignificant except for a larger posterior CC in musicians compared to nonmusicians (*p* = 0.05, one-tailed, 7.3% difference between group means; for details on the analysis see Materials and Methods).

### Functional symmetry

Measures of interhemispheric functional symmetry were obtained by correlating each participant’s fMRI brain responses to music at every voxel with their hemispheric counterparts. This indicates how similar the time courses are for each pair of topographically matched voxels.

#### Musicians vs. nonmusicians

**Fig 1:**
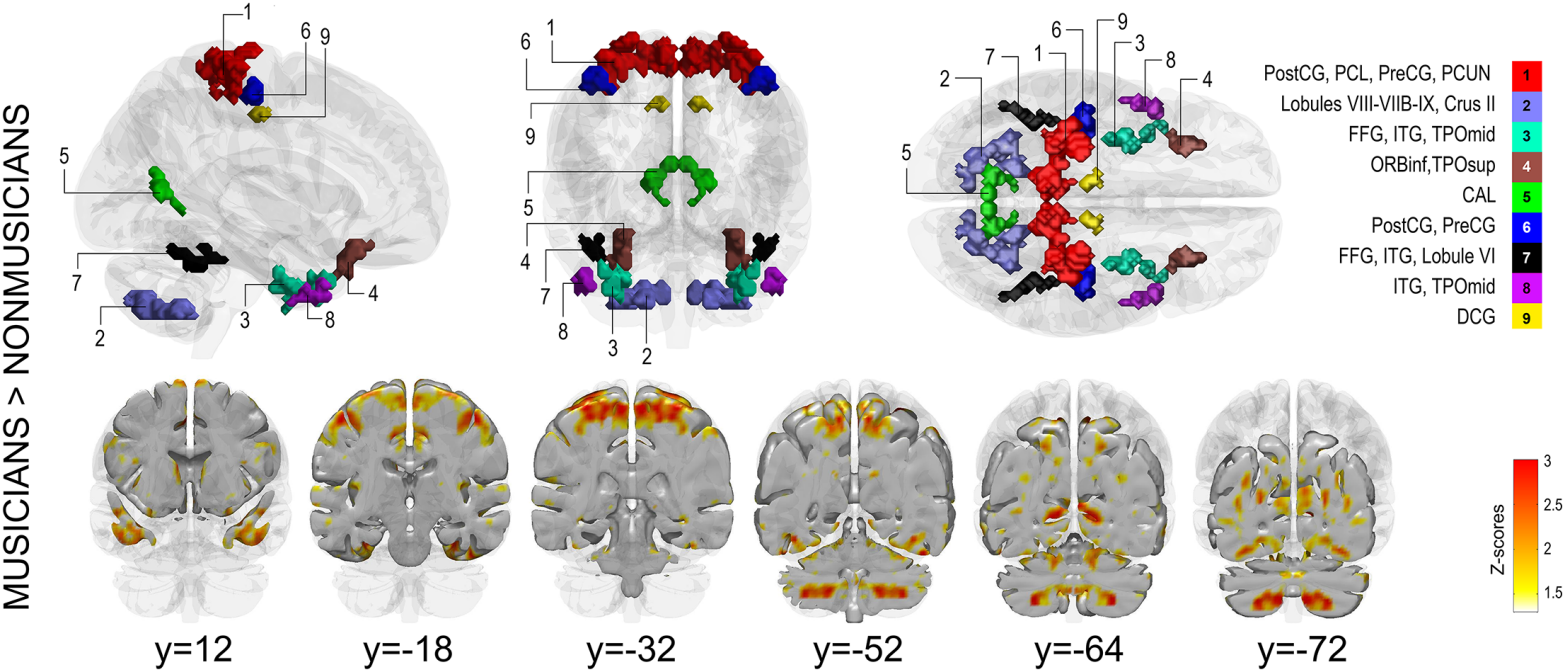
Symmetry maps showing significantly greater functional symmetry for musicians compared to nonmusicians. Top of figure: Orthogonal planes (lateral, frontal, transversal) showing significant clusters (voxelwise thresholded at *p* < 0.01 [z = 2.32]; cluster-wise corrected at *p* < 0.001). Bottom of figure: Coronal slices showing the continuous Z-map for the respective comparison. Abbreviations: PostCG = postcentral gyrus, PCL = paracentral lobule, PreCG = precentral gyrus, PCUN = precuneus, FFG = fusiform gyrus, ITG = inferior temporal gyrus, TPOmid = temporal pole (middle temporal gyrus), ORBinf = orbitofrontal cortex (inferior frontal gyrus), TPOsup = temporal pole (superior temporal gyrus), CAL = calcarine fissure and surrounding cortex, DCG = median cingulate and paracingulate gyrus.

Musicians showed significantly more symmetrical responses to music listening (brain volume = 21.42 cm^3^; brain volumes here refer to the amount of significant voxels, expressed in cm^3^, resulting from the t-test) than nonmusicians (brain volume = 0 cm^3^; one-tailed t-test, *p* < 0.01; see Fig 1 and Table 3 for a list of regions). The symmetry was evident over a widely distributed brain area, including somatomotor regions (paracentral lobule and pre- and postcentral gyri), occipitoparietal lobe (calcarine fissure, precuneus), temporal areas (inferior/superior temporal gyrus [ITG, STG]), prefrontal cortical (PFC) areas (orbitofrontal cortex [OFC]), cerebellum (lobules VI-VIII-VIIIB-IX, Crus II), temporal regions (inferior temporal gyrus [ITG], fusiform gyrus), and a small area in the median cingulate gyrus. Nonmusicians did not exhibit more symmetrical brain responses in any areas than musicians at the chosen significance level. Effect sizes were computed for all voxels. Large effect sizes were found more extensively for musicians (Cohen’s d > 0.8 = 39.26 cm^3^ brain volume) than for nonmusicians (Cohen’s d > 0.8 = 2.82 cm^3^ brain volume).

**Table 3:** Functional symmetry results for musicians.

MUSICIANS	k	max Z	x	y	z	BA
Cluster 1						
Postcentral gyrus	217	3.39	-18	-36	68	4
Paracentral lobule	99	3.55	-8	-36	70	4
Precentral gyrus	97	3.13	-30	-24	74	4
Precuneus	79	3.40	-16	-38	68	4
Cluster 2						
Lobule VIII of cerebellum	153	3.49	-28	-64	-48	-
Lobule VIIB of cerebellum	82	3.56	-20	-70	-42	-
Lobule IX of cerebellum	29	3.12	-16	-48	-50	-
Crus II of cerebellum	12	3.24	-24	-74	-46	-
Cluster 3						
Fusiform gyrus	54	3.05	-28	0	-38	36
Inferior temporal gyrus	39	3.01	-34	-2	-36	36
Temporal pole, middle temporal gyrus	32	2.80	-40	14	-32	38
Cluster 4						
Inferior frontal gyrus, orbital part	69	3.54	-30	26	-18	47
Temporal pole, superior temporal gyrus	24	3.08	-28	24	-30	38
Cluster 5						
Calcarine fissure and surrounding cortex	76	3.37	-14	-64	8	17
Cluster 6						
Postcentral gyrus	54	3.18	-46	-22	54	3
Precentral gyrus	26	2.80	-42	-20	58	4
Cluster 7						
Fusiform gyrus	38	3.56	-44	-50	-24	37
Inferior temporal gyrus	28	3.08	-40	-44	-18	37
Lobule VI of cerebellum	8	2.83	-40	-46	-28	37
Cluster 8						
Inferior temporal gyrus	31	2.96	-52	4	-40	20
Temporal pole, middle temporal gyrus	12	2.76	-48	12	-36	20
Cluster 9						
Median cingulate and paracingulate gyrus	25	2.98	-10	-20	44	-

Brain areas showing significantly greater functional symmetry for musicians compared to nonmusicians. Nonmusicians did not show greater symmetry than musicians. Clusters were obtained via the 18-connectivity scheme employed in SPM. The table reports within-cluster region size (k; i.e., number of voxels), peak Z-statistic value per region within the cluster, and its respective MNI coordinates and Brodmann area (BA). Labels here correspond to the left-hemisphere. Voxels identified as white matter or voxels encroaching very small regions within the cluster (k< 5 voxels) were discarded from the resulting table.

#### Keyboard vs. string players

**Fig 2:**
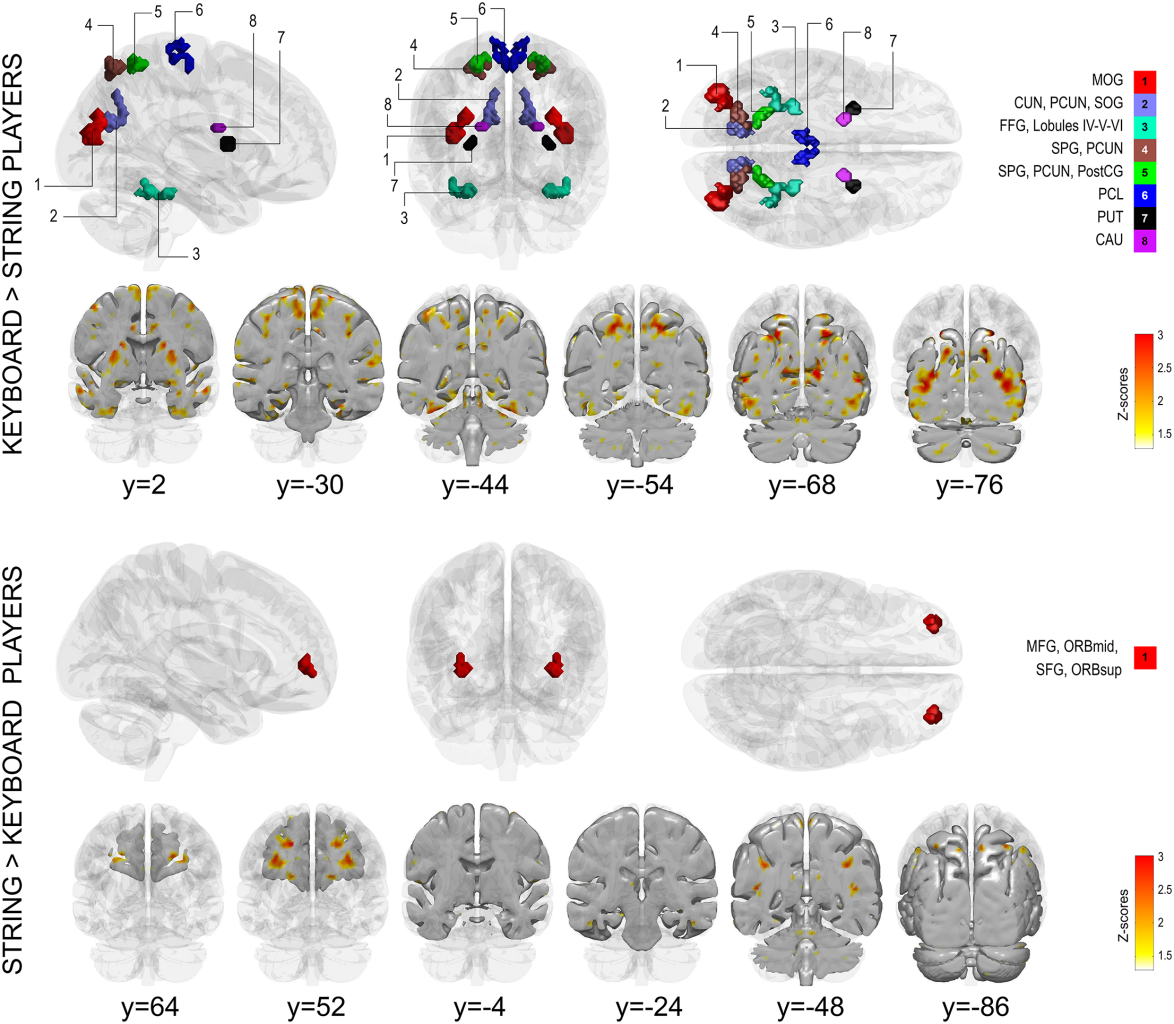
Symmetry maps showing significantly greater functional symmetry for keyboard players compared to string players (top figure) and for string players compared to keyboard players (bottom figure). See legend of Fig 1 for further details. Abbreviations: MOG = middle occipital gyrus, CUN = cuneus, PCUN = Precuneus, SOG, superior occipital gyrus, FFG = fusiform gyrus, SPG = superior parietal gyrus, PostCG = postcentral gyrus, PCL = paracentral lobule, PUT = putamen, CAU = caudate nucleus, MFG = middle frontal gyrus, ORBmid = orbitofrontal cortex (middle frontal gyrus), SFG = superior frontal gyrus, ORBsup = orbitofrontal cortex (superior frontal gyrus).

Keyboardists showed more prominent symmetrical responses to music listening (brain volume = 10.37 cm^3^) than string players (brain volume = 0.90 cm^3^; one-tailed t-test, *p* < 0.01; see Fig 2 [top figure] and Table 4 for a list of regions). Keyboardists’ brain responses were predominantly symmetrical in regions within the occipital and parietal lobes (middle and superior occipital gyrus [MOG, SOG], cuneus, precuneus, superior parietal gyrus ([SPG]), somatosensory cortex (postcentral gyrus), temporal areas (fusiform gyrus), cerebellum (lobules IV-V-VI), and a small subcortical area within the dorsal striatum (caudate nucleus and putamen). For the string players, however, only one small cluster in the middle frontal gyrus (MFG) and SFG displayed more prominent symmetry over the keyboardists (see Fig 2 [bottom figure] and Table 5 for a list of regions). Effect sizes revealed a greater brain volume showing large effect sizes for the keyboardists (Cohen’s d > 0.8 = 184.61 cm^3^) than for the string players (Cohen’s d > 0.8 = 49.31 cm^3^).

**Table 4:** Functional symmetry results for keyboard players.

KEYBOARD PLAYERS	k	max Z	x	y	z	BA
Cluster 1						
Middle occipital gyrus	170	4.55	-36	-84	14	19
Cluster 2						
Cuneus	65	3.76	-14	-70	22	-
Precuneus	26	3.87	-10	-66	40	7
Superior occipital gyrus	10	3.47	-16	-72	22	18
Cluster 3						
Fusiform gyrus	84	3.33	-30	-46	-22	37
Lobule VI of cerebellum	5	3.30	-28	-46	-22	37
Lobules IV-V of cerebellum	5	2.56	-26	-46	-22	37
Cluster 4						
Superior parietal gyrus	83	4.30	-24	-70	58	7
Precuneus	6	3.65	-14	-64	58	7
Cluster 5						
Superior parietal gyrus	50	3.62	-20	-56	60	5
Precuneus	7	2.66	-16	-58	62	5
Postcentral gyrus	5	3.22	-22	-52	58	5
Cluster 6						
Paracentral lobule	59	2.83	-6	-32	62	4
Cluster 7						
Putamen	34	3.55	-28	2	8	48
Cluster 8						
Caudate nucleus	12	2.82	-20	0	20	-

Brain areas showing significantly greater functional symmetry for keyboard players compared to string players (see legend of Table 3 for further details).

**Table 5:** Functional symmetry results for string players.

STRING PLAYERS	k	max Z	x	y	z	BA
Cluster 1						
Middle frontal gyrus	27	2.96	-32	50	6	10
Middle frontal gyrus, orbital part	10	2.78	-32	54	-4	47
Superior frontal gyrus	10	3.28	-30	52	0	11
Superior frontal gyrus, orbital part	9	3.12	-30	54	-2	11

Brain areas showing significantly greater functional symmetry for string players compared to keyboard players (see legend of Table 3 for further details).

## Discussion

### Morphometric analysis of the corpus callosum

When comparing posterior and anterior callosal measures in musicians vs. nonmusicians, and in keyboard vs. string players, only musicians’ posterior callosa were significantly larger compared to those of nonmusicians. Lee and others [18] found a similar effect between musicians and nonmusicians in their morphometric study. However, they found a significant difference in the anterior section of the callosum, while a near-significant trend was observed in the posterior section. Additional correlational analyses were performed between callosal volumes and symmetry indices. However, they did not yield any significant results.

Although there exists evidence linking increased callosal volume, number of fibres crossing through the callosum, and enhanced interhemispheric connectivity [19–21] which suggests that callosal size is a good a marker of information transfer between hemispheres, there does not seem to be a consensus in the literature on a strict correlation between callosal size and the efficiency of interhemispheric transfer, which obscures this relationship [22].

Furthermore, interhemispheric functional connectivity can be widely preserved following callosal agenesis [23] or surgical lesions of the callosum [24–27]. Thus, decreased structural connectivity is not necessarily associated with decreased functional connectivity [25].

Although callosal structure comprises several independently functioning components that under some conditions may produce contralateral inhibition [47], it is widely assumed that the role of the corpus callosum is excitatory. However, the callosum may be a channel for both interhemispheric excitation and inhibition [47]. It is a task for future research to investigate the callosal structures in detail using appropriate methods to better characterize callosal fibres in combination with interhemispheric functional measures [48].

The present non-conclusive result of the morphological analyses of the callosum exposes the lack of agreement in previous neuroimaging results regarding the relationship between callosal size and interhemispheric transfer.

### Functional symmetry

#### Musicians vs. nonmusicians

The increased functional symmetry in musicians, mainly observed in brain regions involved in somatosensory and motor control in the parietal and frontal lobes, is in agreement with the specific motor demands of musicianship. Instrument practice has been shown to enhance motor ability as measured by finger dexterity in both hands [14]. Also the prominent symmetry observed in musicians’ cerebellar responses conforms to the specific motor demands of musicianship. The cerebellum is central to motor programming and learning and therefore play a crucial role in developing musical skills [49]. The intensity of musical training manifests in cerebellar morphology, where cerebellar volume and lifelong intensity of practice correlate positively [16].

Rather than being confined only to motor and perceptual processes, the demands of musicianship are complex and multimodal, supported by several skills developed during years of study [50]. These include bottom-up skills such as the ability to perceive and distinguish the physical properties of music, and top-down skills such as the ability to predict musical events based on prior musical exposure. Instrument practice seems to enhance auditory melodic and rhythmic discrimination [14]. For instance, musicians react faster than nonmusicians to sound stream presentation, especially to sounds consisting of familiar timbres [51,52], but also to slight mistunings [53], indicating superior attentive auditory discrimination skills for musically trained individuals. They also show a mismatch negativity (MMN) for tones mistimed by only 20 ms compared to nonmusicians [54]. Furthermore, when playing in an ensemble, ensuring tight coordination and prompt responses to several sensory stimuli in the interaction with other team members is crucial for a successful joint performance. Such expert skills may require the symmetric use of both hemispheres for speed and efficiency (e.g., in multimodal integration), reflecting greater functional connectivity between homotopic brain areas. Previous research has revealed a more balanced attentional capacity and faster choice reaction times in musicians, as well as enhanced visuomotor ability, when compared to nonmusicians [55], which was attributed by the authors to the cognitive demands of playing a bimanual instrument from childhood.

We also observed symmetric brain responses in musicians’ fronto-parietal areas belonging to the human mirror neuron system [56]. Listening to music may have hence activated neurons that also govern the motor production of those sounds, extending findings obtained in studies on music listening [57]. We speculate that musical training would shape the symmetry of the brain responses mainly in fronto-parietal regions due to its coupling between production and perception of music. One question that arises is whether the symmetrical motor activity is in response to the music. It is known that the motor system is active in response to music listening [58], and displays significant mean inter-subject correlation bilaterally [59], suggesting that the bilateral motor responses are likely to be stimulus-driven.

#### Keyboard vs. string players

The enhanced somatomotor functional symmetry of the keyboard players over the string players may be understood as a result of a more mirrored use of both hands and fingers in keyboardists than in string players. Furthermore, keyboardists had enhanced functional symmetry in subcortical brain responses from the dorsal striatum (comprising caudate nucleus and putamen). This striatal area is an important hub, receiving input from sensorimotor and association cortices. One of its functions is to mediate inhibition of voluntary fine-motor movements when required [60], and in holding back prepared motor responses [61]. Thus, the different symmetry in keyboard and string players reflects specific competences required for mastering each instrument: a midline, symmetrically bimanual instrument like the piano [28] where exact motor timing for synchronization of both hands is required [62], as opposed to a mediolateral, asymmetrically bimanual instrument like the violin, in which arms, hands and fingers play a different role when performing, namely, the right hand controls the movement of the bow while the left hand is concerned with fingering the strings, and where the coordination between fingering and bowing is not synchronous [63]. Thus, although playing a string instrument also requires fine motor skills and bimanual hand coordination, it enforces a strict asynchrony between finger movements (placing fingers on the board) and the up and down bowing. This guides the differences between string players and keyboardists, who instead need absolute synchrony between hands to achieve flawless performance.

The prominent symmetry largely focalized in the visual areas in keyboardists compared to string players may arise from the need to acquire visual information (i.e., score reading) for both right and left hands, while simultaneously monitoring the synchronized movements of both hands. Piano playing from a score is a complex transcription task with high-visual-load activity that involves active, continuous, multiple-part reading of parallel sequences of events. This requires efficient visual scanning strategies [64]. In contrast, score reading for string players is for the most part a serial process, that is, the reading of one melody line at a time. The implications of these unequal visual-processing requirements may have an impact on the interhemispheric synchrony and speed of the visual responses, which, in turn, would affect the degree of functional symmetry in the implicated areas, as observed in the present study. The observed functional symmetry in visual areas in musicians (specifically keyboardists) is in agreement with work by Patson and others [28]. They observed an unusual lack of asymmetry in the interhemispheric transfer time and latency of the visual responses of musicians vs. nonmusicians. In other words, their results indicated a more balanced visual processing in musicians than in nonmusicians. Since in their study most of the musicians played a midline, bimanual instrument (i.e., piano or clarinet), they hypothesized that the cognitive demands of such instruments, and particularly the transfer of visual inputs from musical scores to bilateral motor outputs, may produce equilateral neural connectivity and myelination in both hemispheres, advantageous for speed and accuracy in musical performance. Our additional finding of significantly stronger symmetry of string vs. keyboard players in the MFG/SFG is a novel one and calls for further study.

These results are meaningful in the light of a recent study by Vollman and others [65] which evidenced how different instrument training regimes may result in different structure-function relationships. They observed that string players exhibited a significant positive relationship between fractional anisotropy, a measure of white matter organization, in the posterior midbody of the corpus callosum, and interhemispheric inhibition (IHI), as examined by transcranial magnetic stimulation. Interestingly, this relationship was not significant in pianists or in non-expert controls. The microstructural white matter architecture of the corpus callosum was thus assessed as a marker for interhemispheric information processing within the motor system, replicating previous results in the literature, namely, that microstructural information of the hand callosal motor fibres significantly correlates with functional connectivity measures of IHI between the primary motor cortical hand areas in both hemispheres [66]. These findings indicate the existence of a link between the mode of bimanual training (piano vs. string players), neurophysiology and brain anatomy, as seen in the white-matter structure in the corpus callosum. Consequently, the characterization of the white matter tracts, rather than the size of the corpus callosum, may better reflect a correlation with interhemispheric functional connectivity measures.

Increased transfer of information across hemispheres does not necessarily result in enhanced functional symmetry, unless it happens between anatomically equivalent areas. Thus the condition of homotopicity needs to be satisfied. Functional brain asymmetries as a result of violin practice (e.g., left-hand finger motor specialization [8]) render homotopic brain regions not anatomically or functionally equivalent. This would explain why string players showed significantly less symmetry than keyboardists in motor areas. Thus practice-dependent brain plasticity seems to be a potential factor in the emergence of different symmetry patterns between groups.

The increased symmetric responses in motor and visual areas in musicians, particularly in keyboard players, are assumed to derive from the intensive practice of symmetrical bimanual movements and multipart reading. This would support the premise that functional symmetry results from interhemispheric (thus transcallosal), rather than intrahemispheric, integration. Thus, although functional symmetry could result from strengthened auditory-motor ipsilateral connectivity, our results suggest that it occurs via contralateral connectivity. Moreover, there seems to exists a consensus about the existence of training-induced plasticity in cross-hemispheric connections in musicians, whereas findings on differences in intra-hemispheric fibres between musically trained and untrained individuals have not always been replicated [67].

In view of our present findings on functional symmetry, distinctive kinematics and posture of performing the instrument seem to be crucial factors in shaping the symmetry, although the direction of the effect cannot be inferred from the data. Nonetheless, several studies have found that long-term and intensive musical training may enhance the ability to integrate input from several sensory modalities [14,15,68], which in turn, we hypothesize, may increase the degree of functional symmetry between hemispheres for specific modalities.

#### Limitations

Our study does not directly assess whether increased symmetry is produced by musical training. Previous research has established a positive correlation between brain function, morphology, and early commencement of musical training [6–8,10,15–18,69]. However, whether it is an innate neuroarchitecture that induces functional plasticity predisposing children to thrive musically, or whether the differential neuroarchitecture is an effect of the functional requirements of a life-long, intensive musical training, has to date not been established with a longitudinal study. Many significant aspects seem to influence children’s musical instrument choices, such as sociocultural influences, gender stereotyping, instrument size or timbre, and instrument availability and cost [70]. Thus, although preselectional bias cannot be completely ruled out, functional symmetry differences between keyboard and string players would seem to arise as a result of adaptation to intensive musical training rather than as result of an intrinsic early predisposition [71].

Another limitation of the study is the lack of a behavioral task to support the claim of a cross-modal transfer effect, given that musical training comprises both listening and performing. We argue that the listening side of the musical training is unavoidably influenced or coupled by the motor training resulting from playing an instrument, and this would manifest in the brain responses to music listening alone.

Similarly, one could argue that musicians demonstrated a higher degree of coupling in bilateral motor areas because they were performing motor imagery when listening (i.e. imagining themselves playing the piece), and hence the same could have been potentially true for nonmusicians had they been asked to perform motor imagery. However, if the musical training is driving the coupling of the perceptual-motor system, one could speculate that the degree of homotopic connectivity would be weaker in musically untrained individuals, even if they are asked to perform motor imagery during the listening. These hypothesis-generating results provide a foundation for future studies.

### Conclusion

We show here functional symmetry differences during music listening between musicians and nonmusicians, in addition to functional symmetry profiles for different kinds of musicians [49], thereby demonstrating a dependency between musical training and functional symmetry. Our results indicate a cross-modal transfer effect between musical training and music perception: symmetrical actions derived from musical training manifest in symmetrical brain responses while listening to music. The observed cross-modal transfer of symmetry from sensorimotor to perceptual processing systems suggests that motor training affects music perception. This finding has major implications for a better understanding of cross-modal neuroplasticity, in other words, changes in neural processing in one modality driven by experience or training in another modality [2], an area of increasing interest in neuroscience [50], which investigates the ability of the brain to reconfigure itself to create alternate neural pathways.

## Supporting Information

S1 Information Navigable 3D volume corresponding to Fig 1. Brain areas showing increased functional symmetry in musicians compared to nonmusicians).
(NII)

S2 Information Navigable 3D volume corresponding to top of Fig 2. Brain areas showing increased functional symmetry in keyboard players compared to string players).
(NII)

S3 Information Navigable 3D volume corresponding to bottom of Fig 2. Brain areas showing increased functional symmetry in string players compared to keyboard players).
(NII)
